# The effectiveness and safety of prophylactic central neck dissection in clinically node-negative papillary thyroid carcinoma patients: A meta-analysis

**DOI:** 10.3389/fendo.2022.1094012

**Published:** 2023-01-17

**Authors:** Yao Wang, Yibo Xiao, Yan Pan, Shuhao Yang, Kainan Li, Wei Zhao, Xulin Hu

**Affiliations:** Clinical Medical College & Affiliated Hospital of Chengdu University, Chengdu University, Chengdu, Sichuan, China

**Keywords:** papillary thyroid carcinomas, lymph node excisions, effectiveness, safety, meta-analysis

## Abstract

**Objective:**

This meta-analysis was performed to evaluate the effectiveness and safety of prophylactic central neck dissection (PCND) in patients with clinically node-negative (cN0) papillary thyroid carcinoma.

**Materials and methods:**

A meta-analysis of the literature was performed using the key words “papillary thyroid carcinomas” and “lymph node ecisions” for searches of electronic databases. Complications such as transient hypocalcemia, permanent hypocalcemia, transient and permanent hypoparathyroidism, transient and permanent vocal cord paralysis, transient recurrent and permanent recurrent laryngeal nerve injury, and local recurrence were pooled by meta-analysis. Stata17.0 was used to carry out the meta-analysis.

**Results:**

Data were extracted from 15 studies. In the present review, the group of patients who had total thyroidectomy (TT) with PCND had a lower local recurrence than the group with TT alone (OR 0.22, 95% CI 0.10-0.45, P = 0.000), whereas the incidence of permanent hypocalcemia (OR 4.24, 95% CI 1.05-17.22, P = 0.043) and transient hypoparathyroidism (OR 2.14, 95% CI 1.34-3.42, P =0.001) were higher. No significant differences were recorded in the incidence of other complications: transient hypocalcemia (OR 2.24, 95% CI 0.77-6.51, P = 0.138), permanent hypoparathyroidism (OR 1.70, 95% CI 0.89-3.27, P = 0.111), transient vocal cord paralysis (OR 1.48, 95% CI 0.78-2.83, P = 0.231), permanent vocal cord paralysis (OR 1.44, 95% CI 0.53-3.94, P = 0.477), transient recurrent laryngeal nerve injury (OR 1.47, 95% CI 0.93-2.32, P = 0.102) and permanent recurrent laryngeal nerve injury (OR 1.24, 95% CI 0.56-2.74, P = 0.587) between the two groups.

**Conclusion:**

Compared with TT alone, TT with PCND was more effective in reducing local recurrence without increasing the risk of recurrent laryngeal nerve, thyroid and vocal cord, except for hypocalcemia and transient hypoparathyroidism. Therefore, we believe that TT with PCND should be recommended for patients with cN0 PTC.

**Systematic review registration:**

https://www.crd.york.ac.uk/prospero/, identifier CRD4202 2355078.

## Introduction

The incidence of papillary thyroid carcinoma (PTC) continues to increase (1, 2). PTC is the most common type of thyroid malignancy and is characterized by a well-differentiated, slow-growing and high rate of lymph node metastasis. Surgical resection is the best treatment when visible nodules are present (3–5). Thyroid with isthmus subtotal resection or total resection is determined by the thyroid disease of patients. Total thyroidectomy (TT) can effectively avoid the occurrence of residual metastases in the gland, especially for differentiated thyroid cancer. However, this operation also has several shortcomings. Once the patient relapses, a second surgery is needed. The metastasis rate of central lymph nodes is high in patients with stage C node-negative (N0) PTC. Therefore, some scholars have proposed that the treatment of this disease can be combined with central lymph node dissection to reduce the risk of postoperative metastasis and recurrence and to avoid long-term secondary surgical dissection of central lymph nodes (6). To date, PCND have be considered in patients with PTC with clinically uninvolved central neck lymph nodes who have advanced primary tumors (T3 or T4) (5), there has been debate on whether to conduct prophylactic central lymph node neck dissection (PCND) for clinically node-negative (cN0) patients (7–11). Reasons to omit PCND are listed as follows: (1) no survival benefit, (2) increasing the risk of recurrence and postoperative complications, and (3) associated with longer hospitalization times and additional costs (12). Thus, a meta-analysis was conducted to provide strong evidence for clinical practice by assessing the effectiveness and safety of TT with PCND in cN0 PTC patients.

## Materials and methods

### Search strategy

We systematically searched the PubMed database from its establishment to June 2022 with the following keywords: “papillary thyroid carcinomas” and “lymph node excisions”. All identified articles were hand-searched.

### Selection of articles

We screened articles according to the following criteria. (1) The subjects were patients with cN0 PTC. (2) The study was designed as a retrospective or prospective controlled clinical trial. (3) Patients in the experimental group received TT with PCND, and the control group received TT alone. (4) The article provided complete information such as complications, features and number of subjects. (5) The diagnostic criteria and therapeutic evaluation indexes were displayed.

Studies were excluded from our meta-analysis for the following reasons: duplicate articles, unavailable data, only abstract available, and nonclinical publications.

### Data extraction

The data were extracted according to the following information: (1) The first author, publication time and sources. (2) Study type. (3) Number of subjects, features and therapeutic effect. (4) Results. If there were any controversies between the two researchers, the final decision was made by a third researcher.

### Statistical analysis and quality assessment

Based on the preset protocol registered with PROSPERO 2022 (CRD4202 2355078) and the guidelines of the preferred reporting items for systematic reviews and meta-analyses (PRISMA) statement, we performed our study (13). Stata 17.0 was used for meta-analysis. Standard deviation (SMD) with 95% confidence interval (CI) was used to calculate the continuous variables, while odds ratio (ORs) with 95% CI was used to calculate dichotomous variables. A random-effects model was used to estimate the pooled effects. The significance level was 0.05. The inconsistency test (I^2^) was used to assess heterogeneity when I^2^>50% was considered high heterogeneity. Publication bias was estimated by Begg’s and Egger’s tests. The Newcastle−Ottawa scale (NOS) was used for the assessment of retrospective studies. Cochrane’s Risk of Bias 2 (RoB2) tool was used for assessment of randomized controlled trials (RCTs). When discussions ran into disagreement, consensus was reached with another member.

## Results

**Figure 1:**
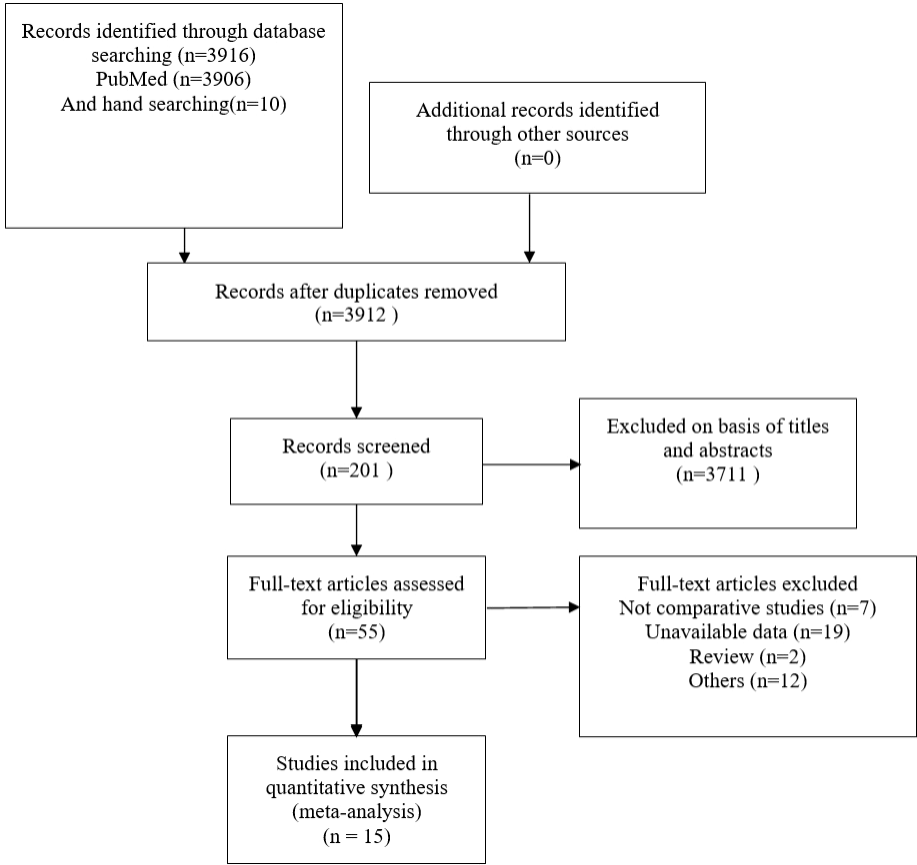
Identification of eligible articles.

**Figure 2:**
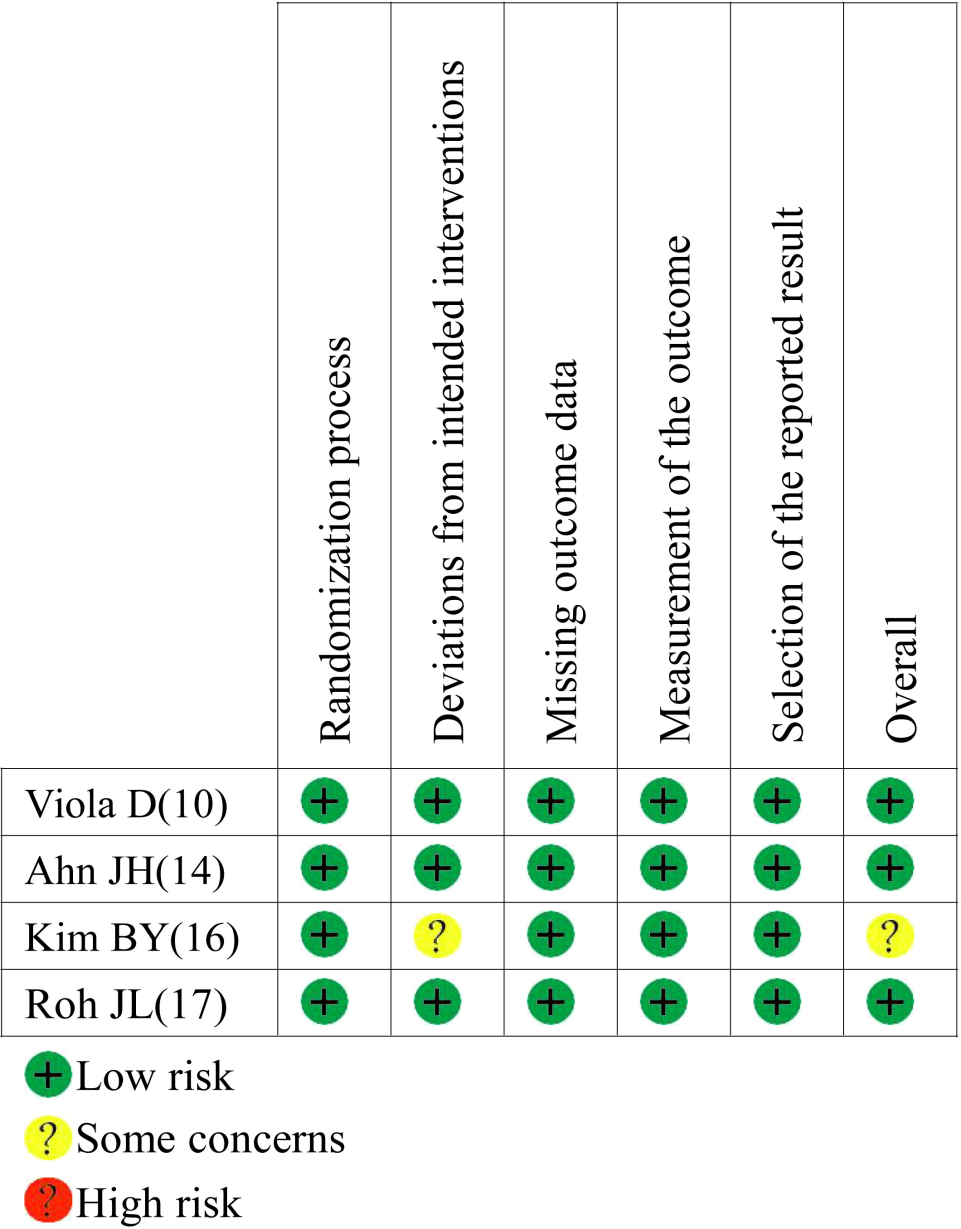
Risk of bias assessment for RCTs.

A total of 3916 publications were obtained from PubMed and 10 from hand searches. After 40 inappropriate articles were excluded, 15 studies (7, 9–12, 14–23) were eligible and incorporated into our study (**Figure 1**). The basic characteristics of the included studies are shown in **Table 1**. The clinical outcomes of the included studies are shown in **Table 2**. There were 11 retrospective studies and 4 RCTs. All the included studies demonstrated a relatively high quality, and the results are shown in **Supplementary Table 1** and **Figure 2**. No publication bias was found, and the results of Begg’s and Egger’s tests are shown in **Supplementary Figure 1**.

**Table 1:** Basic characteristics of included studies.

The first author	Published year	Country	Case size	TT with PCND	TT alone	Included indexes
Ahn JH (14)	2021	Korea	101	51	50	⑦⑨
Barczynski M (7)	2013	Germany	640	358	282	⑦⑧⑨⑩
Calò PG (22)	2017	Italy	133	30	103	⑥⑨⑩
Conzo G (9)	2014	Italy	752	362	390	③④⑨⑩
Dobrinja C (19)	2017	Italy	186	74	112	⑦⑧⑨⑩
Hartl DM (18)	2013	France	246	155	91	⑥⑧⑩
Hyun SM (11)	2012	Korea	152	65	87	③⑤⑥
Jin SH (21)	2020	Germany	108	54	54	③⑤⑥⑨
Kim BY (16)	2019	Korea	164	82	82	③⑥
Korkmaz MH (23)	2017	Turkey	302	162	140	⑧⑨⑩
Moo TA (20)	2010	America	81	45	36	⑥⑦⑨⑩
Roh JL (17)	2007	Korea	155	82	73	①②③④
So YK (15)	2012	Korea	232	119	113	①②③④
Viola D (10)	2015	Italy	181	93	88	⑩
Yazici D (12)	2020	Germany	358	258	100	⑤⑥⑦⑧⑨⑩

Observation indexes: ①Transient hypocalcemia;②Permanent hypocalcemia;③Transient vocal cord paralysis;④Permanent vocal cord paralysis;⑤Extrathyroid extension;⑥ local recurrence;⑦Transient recurrent laryngeal never injury;⑧Permanent recurrent laryngeal nerve injury; ⑨Transient hypoparathyroidism; ⑩Permanent hypoparathyroidism.

**Table 2:** Comparison of outcomes between TT with PCND group and TT alone group.

The first author	local recurrence	Transient hypocalcemia	Permanent hypocalcemia	Transient vocal cord paralysis	Permanent vocal cord paralysis	Transient recurrent laryngeal never injury	Permanent recurrent laryngeal nerve injury	Transient hypoparathyroidism	Permanent hypoparathyroidism
Ahn JH (14)	NR	NR	NR	NR	NR	5/3	NR	7/13	NR
Barczynski M (7)	NR	NR	NR	NR	NR	26/18	9/6	109/37	8/2
Calò PG (22)	0/4	NR	NR	NR	NR	NR	NR	7/13	1/2
Conzo G (9)	NR	NR	NR	13/5	6/3	NR	NR	53/27	13/4
Dobrinja C (19)	NR	NR	NR	NR	NR	7/3	3/1	11/9	6/1
Hartl DM (18)	3/11	NR	NR	NR	NR	NR	2/2	NR	6/4
Hyun SM (11)	1/13	NR	NR	1/0	NR	NR	NR	NR	NR
Jin SH (11)	2/4	NR	NR	1/3	NR	NR	NR	4/5	NR
Kim BY (16)	3/1	NR	NR	0/1	NR	NR	NR	NR	NR
Korkmaz MH (23)	NR	NR	NR	NR	NR	NR	2/0	22/17	6/5
Moo TA (20)	2/6	NR	NR	NR	NR	2/0	NR	31/5	0/5
Roh JL (17)	NR	25/7	4/0	6/3	3/2	NR	NR	NR	NR
So YK (15)	NR	49/38	7/2	4/5	1/2	NR	NR	NR	NR
Viola D (10)	NR	NR	NR	NR	NR	NR	NR	NR	7/18
Yazici D (12)	7/19	NR	NR	NR	NR	16/5	1/1	69/10	3/2

Values are all given as TT with PCND group/TT alone group. NR, not report.

### Efficacy

#### Local recurrence

**Figure 3:**
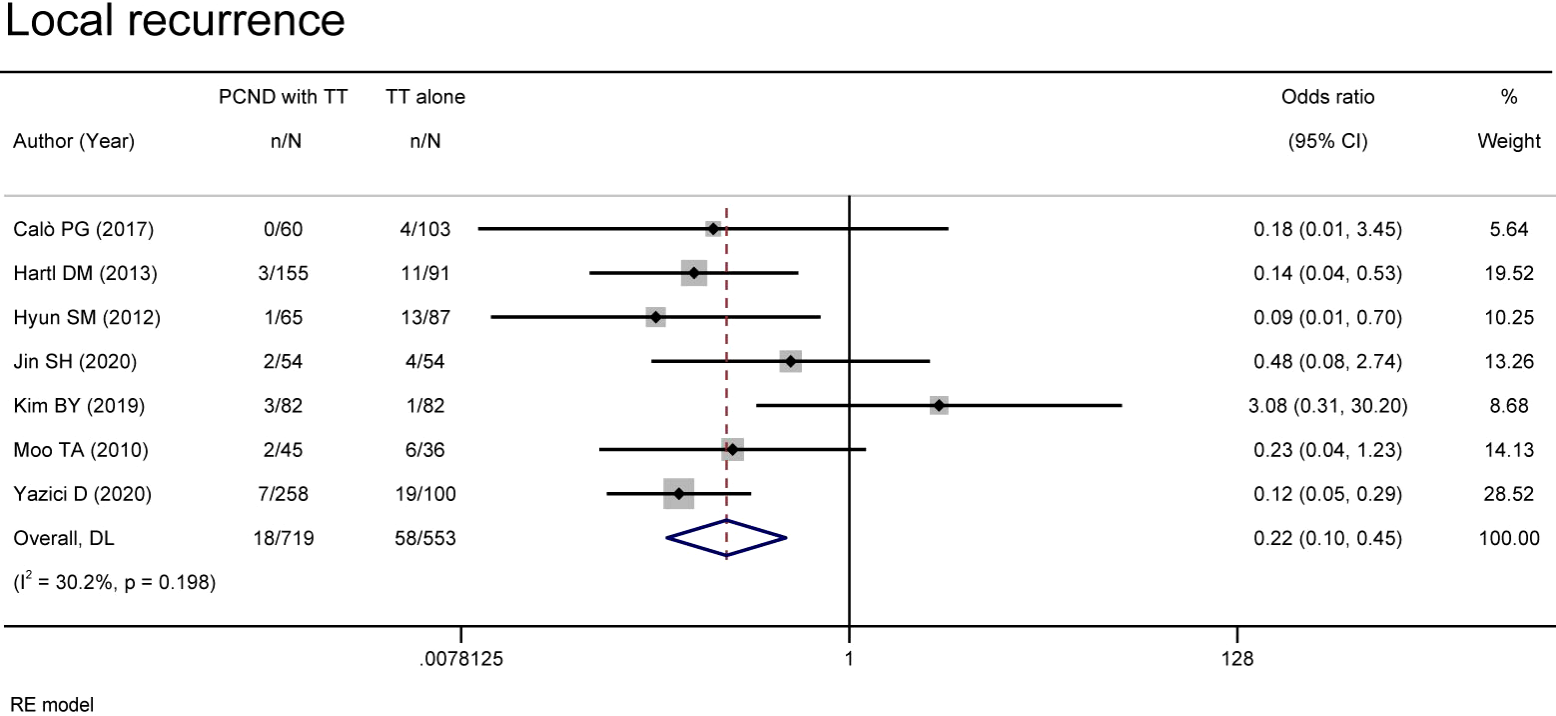
Forest plot of local recurrence in TT with PCND and TT alone groups.

Local recurrence was reported in 7 studies (11, 12, 16, 18, 20–22). A random effects model suggested that the group of TT with PCND had a lower recurrence rate than the group of TT alone (OR 0.22, 95% CI 0.10-0.45, P = 0.00). There was no significant heterogeneity between studies (I² = 30.2%, P = 0.198) (**Figure 3**).

### Safety

#### Transient hypocalcemia

**Figure 4:**
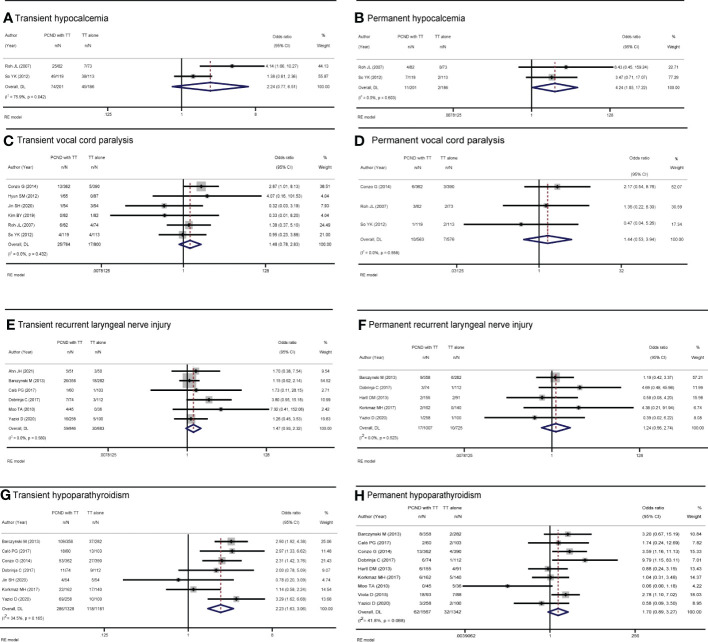
Forest plot of **(A)** transient hypocalcemia, **(B)** permanent hypocalcemia, **(C)** transient vocal cord paralysis, **(D)** permanent vocal cord paralysis, **(E)** transient recurrent laryngeal nerve injury, **(F)** permanent recurrent laryngeal nerve injury, **(G)** transient hypoparathyroidism, and **(H)** permanent hypoparathyroidism in TT with PCND and TT alone groups.

Of the 15 studies, 2 studies (15, 17) reported transient hypocalcemia, but there was no significant difference between the two groups (OR 2.24, 95% CI 0.77-6.51, I² = 75.9%, P = 0.138) (**Figure 4A**).

### Permanent hypocalcemia

Two studies (15, 17) reported permanent hypocalcemia. A random-effects model indicated that the incidence of permanent hypocalcemia in the TT with PCND group was higher than that in the TT alone group (OR 4.24, 95% CI 1.05-17.22, P = 0.043). No heterogeneity was recorded in our studies (I² = 0, P = 0.603) (**Figure 4B**).

### Transient vocal cord paralysis

Transient vocal cord paralysis was discussed in 6 studies (9, 11, 15–17, 21). The incidence of transient vocal cord paralysis was not significantly different between the TT with PCND group and the TT alone group (OR 1.48, 95% CI 0.78-2.83, I² = 0, P = 0.231) (**Figure 4C**).

### Permanent vocal cord paralysis

There studies (9, 15, 17) reported permanent vocal cord paralysis, but no significant difference was found between the two operated groups (OR 1.44, 95% CI 0.53-3.94, I^2^ = 0, P = 0.477) (**Figure 4D**).

### Transient recurrent laryngeal nerve injury

Six studies (7, 12, 14, 19, 20, 22) reported transient recurrent laryngeal nerve injury, and there was no significant difference between the TT with PCND group and the TT alone group (OR 1.47, 95% CI 0.93-2.32, I² = 0. P = 0.102). (**Figure 4E**).

### Permanent recurrent laryngeal nerve injury

Permanent recurrent laryngeal nerve injury was reported in five studies (7, 12, 18, 19, 23). There was no significant difference between the TT with PCND group and the TT alone group (OR 1.24, 95% CI 0.56-2.74, P = 0.587). No heterogeneity was recorded in these studies (I^2^ = 0, P = 0.523). (**Figure 4F**).

### Transient hypoparathyroidism

Nine included studies (7, 9, 12, 14, 19–23) reported transient hypoparathyroidism. The incidence of transient hypoparathyroidism in the TT with PCND group was higher than that in the TT alone group (OR 2.14, 95% CI 1.34-3.42, I^2^ = 71.9%, P = 0.001). Sensitivity analysis suggested that the studies of Ahn et al. (14) and Moo et al. (20) influenced our results, which was caused by the broad criteria for defining transient hypoparathyroidism. In Ahn’s study, the criteria for transient hypoparathyroidism were serum parathyroid hormone levels below baseline, need for oral calcium supplementation or hypocalcemia symptoms (14). In the study of Moo et al, hypoparathyroidism was defined as calcium levels below the normal range (8.5/10.5 mg/dl) or the presence of symptoms of hypocalcemia (20). This may be one of the sources of heterogeneity. After omitting the two studies, the pooled result was OR 2.23 (95% CI 1.63-3.06, I^2 =^ 34.5%, P=0.00) (**Figure 4G**).

### Permanent hypoparathyroidism

Nine studies (7, 9, 10, 12, 18–20, 22, 23) reported permanent hypoparathyroidism. The incidence of permanent hypoparathyroidism was not significantly statistic difference in the two groups (OR 1.18, 95% CI 0.53-2.64, I^2^ = 41.8%, P = 0.111). (**Figure 4H**).

## Discussion

In the present meta-analysis, the efficacy of TT with PCND in cN0 PTC patients was shown to be relatively satisfactory. PCND reduced the risk of local recurrence. Several factors might explain our findings. First, thyroid cancer metastasizes through the lymphatic pathway, and the central lymph node is the first stop of thyroid lymphatic reflux. Second, lymph node metastases are often difficult to detect before surgery (24, 25). The completeness of surgical resection is an important determinant of prognosis in patients with PTC (26). PCND improves the accuracy of staging, removes undetected involved lymph nodes, and reduces recurrence in patients with cN0 PTC. Thus, PCND could avoid the metastatic spread of cancer cells and prevent future recurrence (22). However, some studies are inconsistent with our conclusions. Kim, Conzo et al. (9, 16) proposed that PCND did not help regional recurrence and suggested radioactive iodine therapy to control the disease. Conzo et al. (9) showed that reoperations occurred less frequently when an experienced endocrine surgeon performed the procedure. This is in contrast to the findings of Moo et al (20), who found a trend toward lower recurrence in TT with PCND, which could be due to increased control over local metastases or increased doses of radioiodine therapy (RAI) ablation in patients with recognized lymph node metastases. Yazıcı et al. (12) also recommended PCND in cN0 PTC patients, and they found that patients who received TT with PCND had less laryngeal nerve injury recurrence, reoperation and need for RAI therapy than patients who received TT alone.

Regarding safety, while the incidence of permanent hypocalcemia and transient hypoparathyroidism in the TT with PCND group was higher than that in the TT group, other complications were similar between the two groups. Transient hypoparathyroidism is the most common complication after TT. The results of our study showed that the incidence of postoperative transient hypoparathyroidism increased in the TT with PCND group compared with the TT alone group. It is speculated that the structure of the parathyroid glands is tiny and is easily injured or accidentally cut during surgery. Therefore, we recommend that experienced endocrine surgeons perform surgical operations as much as possible, use techniques to accurately locate the parathyroid glands during surgery, and use advanced stripping medical equipment to decrease the risk of accidental debridement and removal of the parathyroid glands.

Moreover, our study showed no significant difference in permanent hypoparathyroidism between the TT alone and TT with PCND groups. However, this result is still controversial. We found that in the study by Moo et al., the number of patients with transient hypoparathyroidism in the TT with PCND group was more than that in the TT alone group at the beginning (20). Some researchers (10, 12, 17, 18) also reached similar conclusions as Moo et al. (20); all of them found that PCND was a risk factor for transient hypothyroidism, while the rate of permanent hypoparathyroidism was very low, and there were no significant differences from patients who received TT alone. In contrast, Dobrinja et al. (19) reported that the incidence of permanent hypothyroidism in the TT with PCND group was significantly higher than that in the TT group, but we found that in this study, the follow-up time of the TT with PCND group was only 37 months, while that of the TT alone group was 76 months. We speculated that this might be because some patients with symptom elimination were not included in the statistics, which caused an increased incidence of permanent hypothyroidism.

Transient hypocalcemia is also a common complication of TT due to the decreases in parathyroidism function (27, 28). Our pooled results showed that no increased risk of postoperative transient hypocalcemia was found in the TT with PCND group compared with the TT alone group. However, our result was inconsistent with some studies. Roh et al. (17) found that the incidence of transient hypocalcemia was higher in the TT with PCND group than in the TT alone group, which was attributed to the fact that expanding lymph node dissection in the process of TT with PCND increases the risk of parathyroid blood vessel resection. In So et al.’s study, the incidence of tumor multifocality was higher in the TT with PCND group than in the TT alone group (15). Overall, the cause of hypocalcemia is related to the scope, mode and frequency of surgery, lymph node dissection, and malignant degree of histopathological examination (29).

Permanent hypocalcemia is defined as the requirement for calcium active vitamin D and/or calcium supplementation within half a year after thyroidectomy, and permanent hypocalcemia requires lifelong treatment (29). Herein, the pooled results of our study showed that the TT with PCND group had a higher risk of permanent hypocalcemia than the TT alone group. We speculated that permanent hypocalcemia may be caused by severe intraoperative parathyroid damage or poor response to treatment of transient hypocalcemia. Moreover, PCND might be a risk factor for permanent hypocalcemia in children (30). Therefore, effective identification and prudent preservation of parathyroid glands and autotransplantation of at least one gland may be a wise move to prevent permanent hypoparathyroidism (31). Careful protection of the vascular supply and parathyroid drainage system during lymph node dissection may reduce the incidence of hypocalcemia (17). Intriguingly, one meta-analysis suggested that the application of intraoperative near-infrared autofluorescence imaging (NIRAF) in thyroidectomy does not increase the risk of hypocalcemia (32). Weng et al. (32) stated that intraoperative NIRAF was used to assist parathyroid imaging and help the surgeon visually localize parathyroid tissue during surgery. It can accurately detect parathyroid glands in almost real time, which could reduce the cost and save time for parathyroid frozen section biopsy. Taken together, the identification and preservation of parathyroid glands should be performed in PTC patients receiving TT with PCND, and using NIRAF to assist in locating thyroid tissue during surgery should be recommended to reduce thyroid blood vessel damage or miscut during lymph node dissection (32). In the case of accidental injury during surgery, parathyroid autotransplantation should be considered.

Additionally, in our meta-analysis, TT with PCND did not increase the incidence of vocal cord paralysis. However, Roh et al. (17) demonstrated an increased risk of transient vocal cord paralysis in TT with PCND, which could be explained by increased recurrent laryngeal nerve injury caused by dissection of the lymph nodes. The same conclusion was found by Hyun et al. (11) They found that the technique of the operator might have contributed to this phenomenon. In contrast, Jin et al. (16) found that the incidence of transient vocal cord paralysis in the PCND with TT group was lower than that in the TT alone group, but permanent vocal cord paralysis did not occur between the two groups. Therefore, we suggest that a skilled surgeon should be selected for PCND.

In the present study, the incidence of recurrent laryngeal nerves was similar among these two groups. It is possible that PCND has the significance of sentinel lymph node biopsy without causing additional damage and could avoid injury to the recurrent laryngeal nerve during neck dissection (33). However, Dobrinja et al. (19) reported that TT with PCND increased the risk of transient recurrent laryngeal nerve injury, whereas permanent recurrent laryngeal nerve injury did not reach statistical significance. This is because PCND means a larger surgical scope and operation time, which increases the chance of recurrent laryngeal nerve injury, but these injuries can be recovered in a period of time after surgery and do not cause permanent recurrent laryngeal nerve injury. Further studies focusing on the mechanisms of the restoration of laryngeal nerve function are still needed.

Another advantage of TT with PCND seems to be the reduction in postoperative triglyceride levels (34). In Korkmaz MH’s study, median postoperative triglyceride levels were higher in the TT alone group (23), which sometimes led to unnecessary testing, increased use of radioiodine therapy, and reoperations (2).

Thyroid surgery is a delicate operation involving a very narrow area. Surgeons not only need to be skilled but also need systematic microsurgical techniques to avoid complications and the risk of reoperation. Anerea et al. (35) noted that microsurgical techniques and magnifying glass magnification enabled us to record the doctor’s enlarged field of view and optimize all anatomical and technical details. These records are an aid to surgical teaching, a training tool for inexperienced surgeons, and a method for self-analysis of surgical techniques (35). Some studies reached similar conclusions that thyroidectomy by an experienced surgeon using microsurgical techniques under an optical magnifying glass significantly reduced the incidence of permanent hypoparathyroidism (36, 37).

It is worth noting that several studies have shown that ipsilateral PCND (Ipsi-PCND) plus frozen section examination (FSE) can be considered an effective alternative to bilateral PCND. Marco and his colleagues noted that FSE was highly accurate in the lymph node status of patients classified as cN0 before and during surgery, and FSE of ipsilateral lymph nodes could be used as a reliable method to evaluate the lymph node status of cN0 patients and determine the scope of central neck dissection during surgery (38–40). In their study, the Ipsi-PCND group had a lower tendency for transient hypocalcemia and a lower complication rate than the TT group. In clinical single-focal cN0 PTC, conventional Ipsi-PCND plus ipsilateral lymph node FSE may be an effective alternative to bilateral PCND, but the risk of contralateral metastasis can be ignored. More clinical trials are needed to confirm the safety and efficacy of Ipsi-PCND.

Several limitations cannot be ignored. First, high-quality RCTs were not included. Second, due to the limitation of sample sizes in different studies, we did not perform subgroup analysis of unilateral and bilateral PCND to determine its impact on complications and local recurrence. Third, we cannot avoid the influence of surgeons in different countries and institutions. Last, the inconsistent criteria for TT with PCND in patients with cN0 PTC in included studies is a weakness of our study.

## Conclusions

From our meta-analysis, we found that TT with PCND in cN0 PTC patients increases the incidence of permanent hypocalcemia and transient hypoparathyroidism while reducing the rate of local recurrence, with no significant difference in other complications compared to TT alone. Thus, we recommend TT with PCND in cN0 PTC patients. Moreover, intraoperative NIRAF is beneficial in reducing the incidence of hypocalcemia. Conventional PCND combined with NIRAF may be a safe and reliable operation and could provide accurate staging for patients with PTC for adjuvant treatment. We recommend that patients go to a more specialized medical center for safe treatment. However, these studies still have some limitations, and more RCTs and prospective studies with long-term follow-up are needed to further confirm the results.

## Data availability statement

The original contributions presented in the study are included in the article/**Supplementary Material**. Further inquiries can be directed to the corresponding author.

## Author contributions

YW and XH originated and designed the study. YW, YX, YP, KL conducted the literature search, data extraction, data analysis and interpretation and drafting of the manuscript. SY and KL conducted the data interpretation and methodology. YW and WZ conducted critical revision of article and final approval. All authors contributed to the article and approved the submitted version.
