# Supplementary material for: The effectiveness and safety of prophylactic central neck dissection in clinically node-negative papillary thyroid carcinoma patients: A meta-analysis
Source: Front Endocrinol (Lausanne). 2023 Jan 17;13:1094012. doi: 10.3389/fendo.2022.1094012 (PMC9886572; doi:10.3389/fendo.2022.1094012)
Supplement: Supplementary file 1 [file DataSheet_1.docx]

**Supplementary Figure 1. The Begg's and Egger's tests.**

**Local recurrence**

**
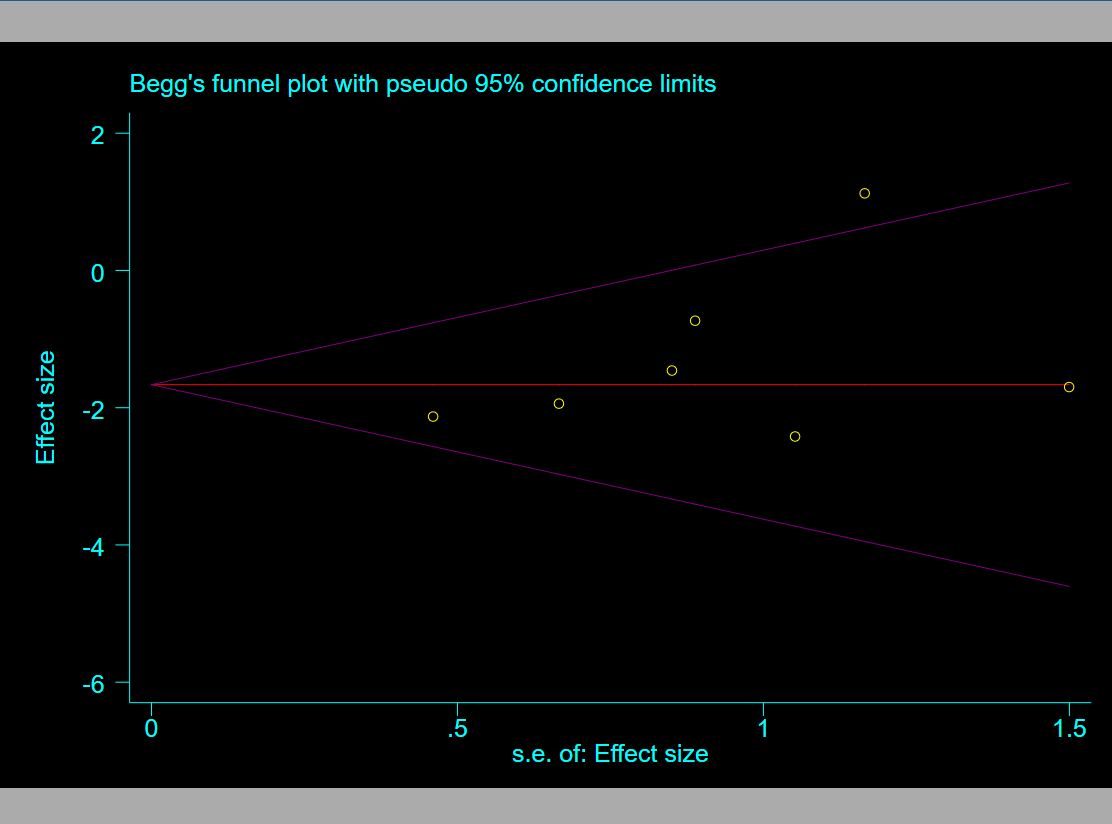

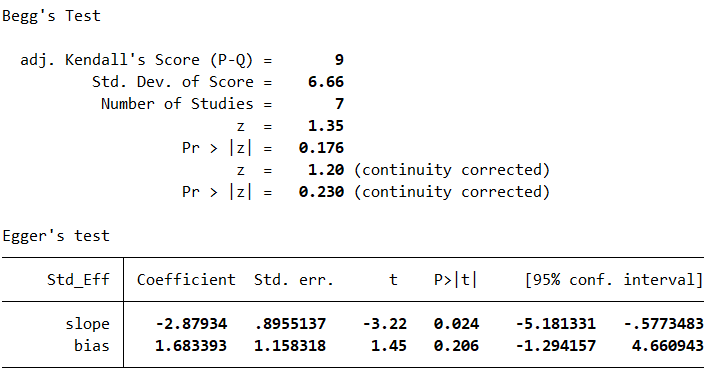
**

**
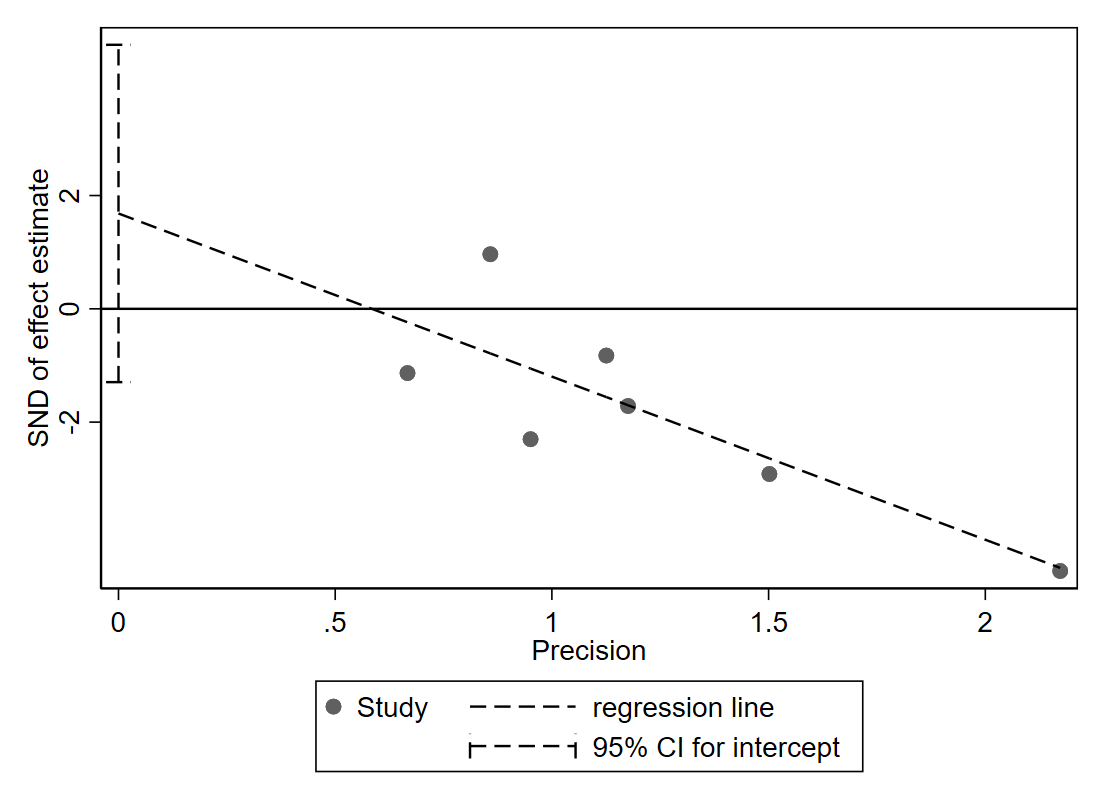
**

**Transient hypocalcemia**

**
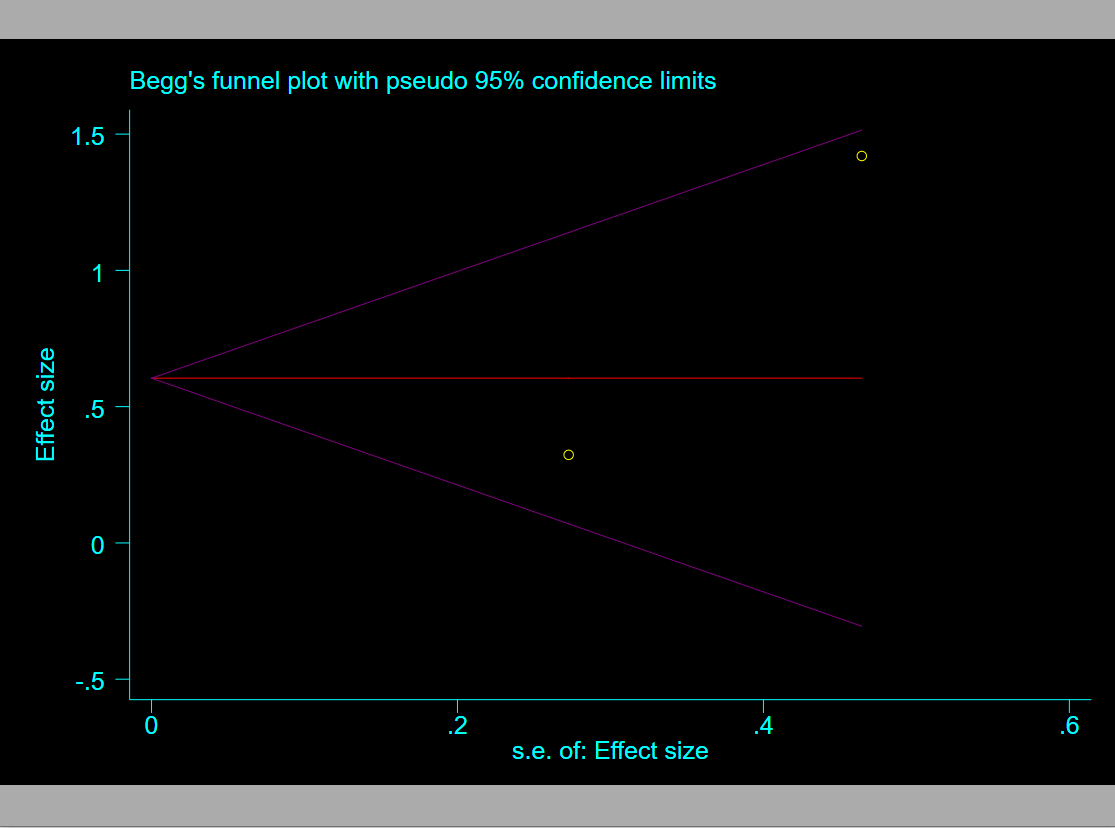

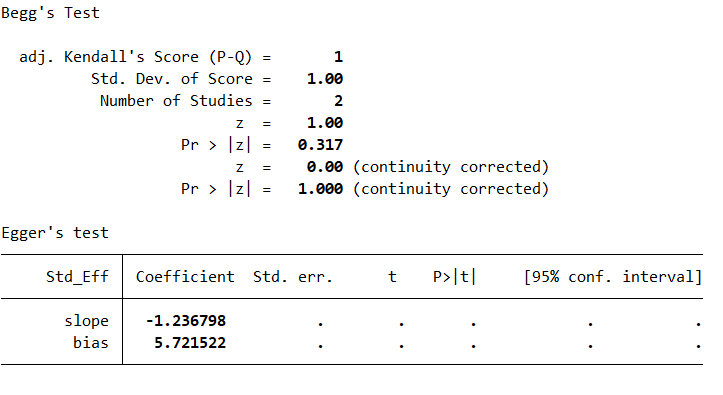

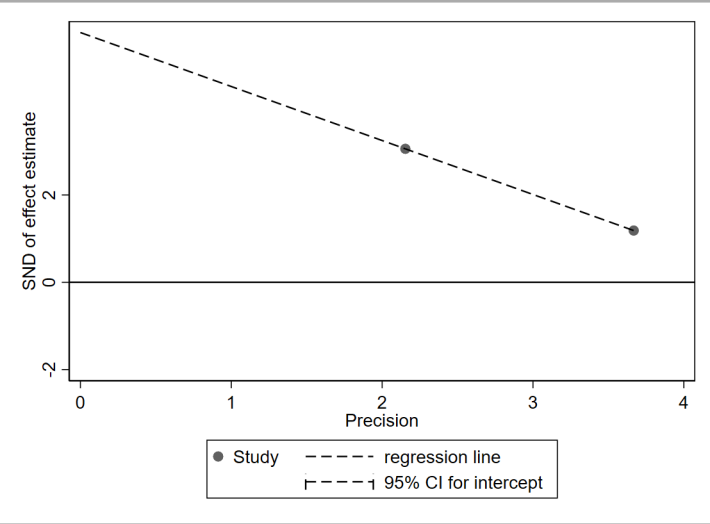
**

**Permanent hypocalcemia**

**
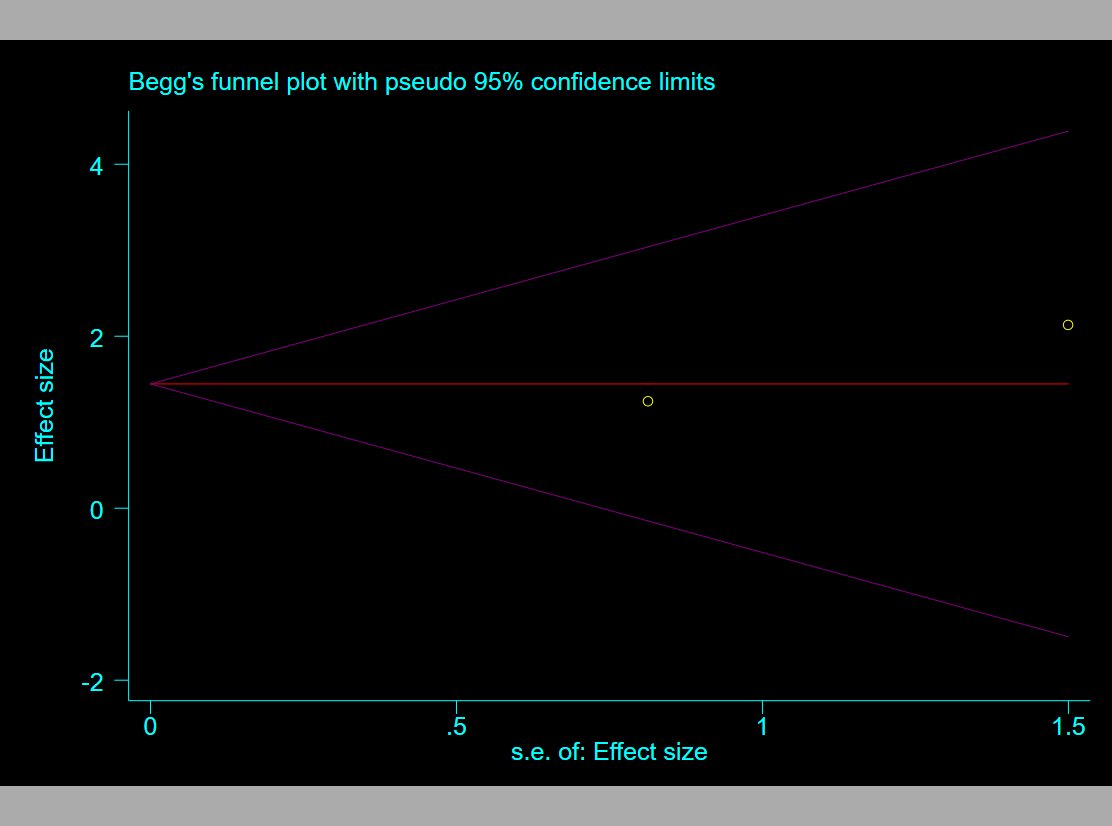
**

**
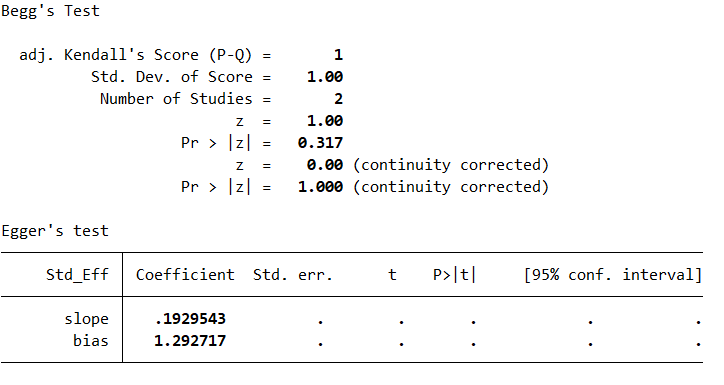
**

**Transient vocal cord paralysis**

**
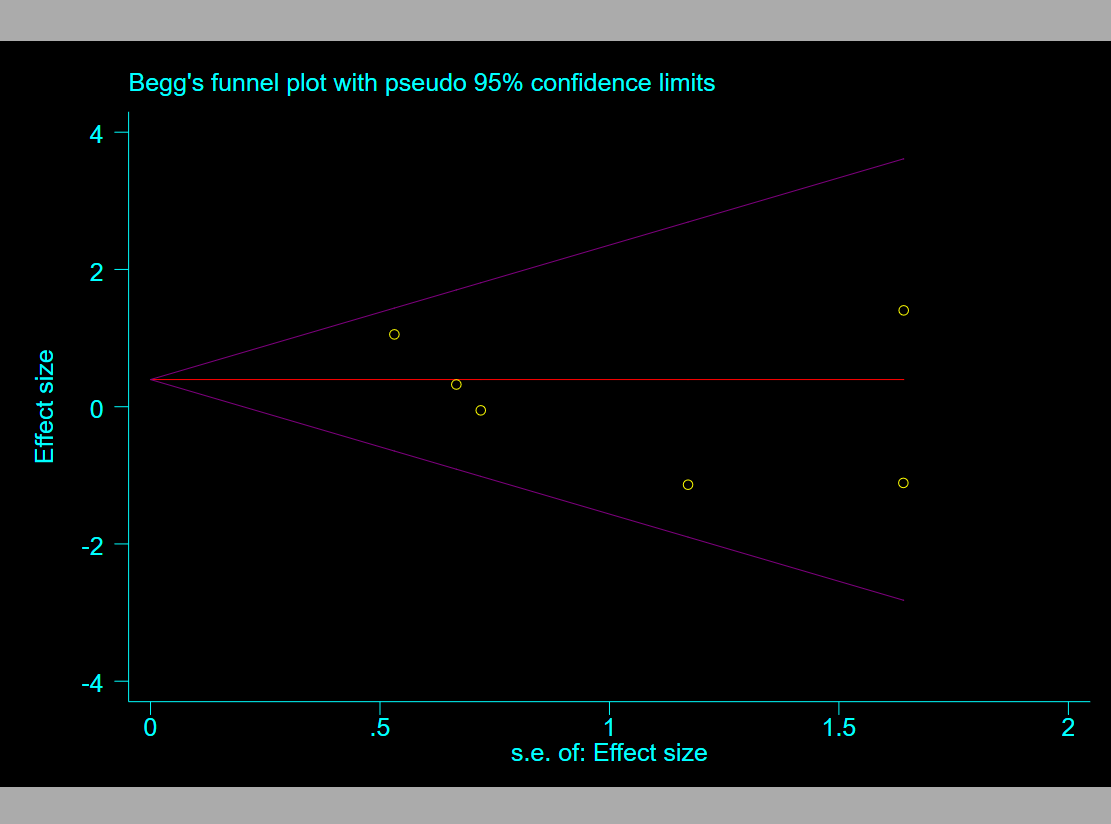

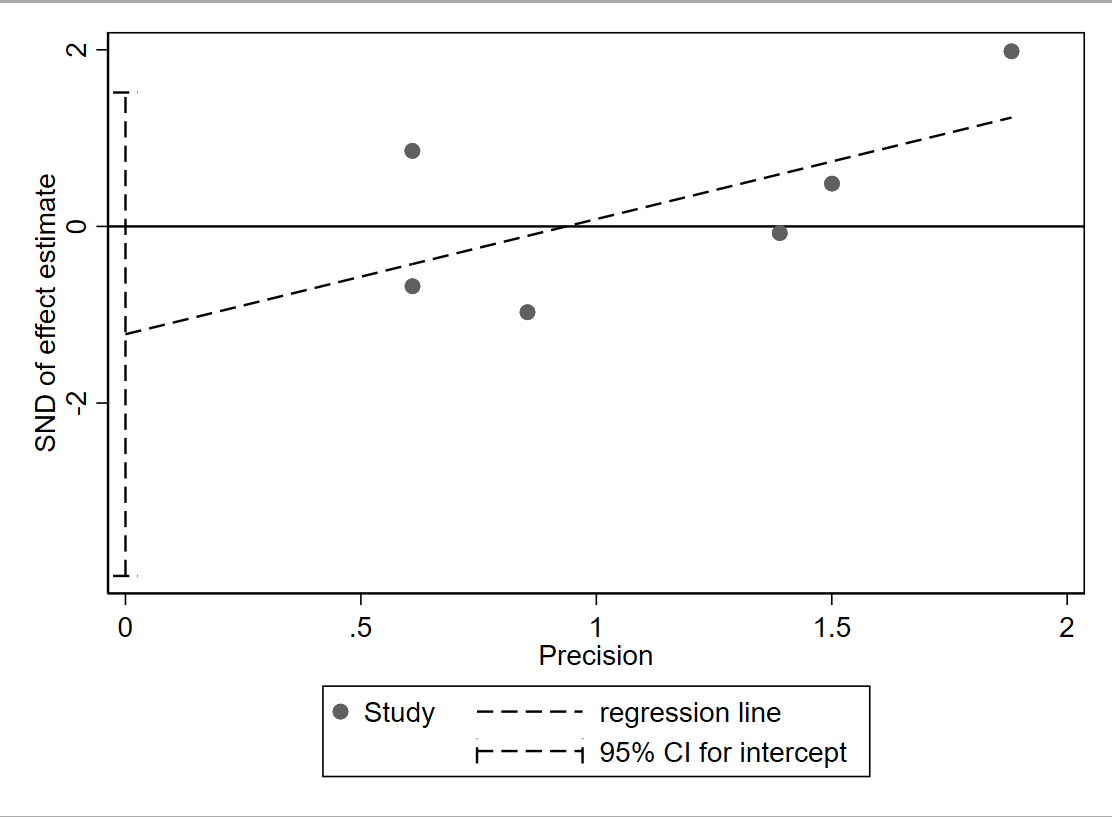

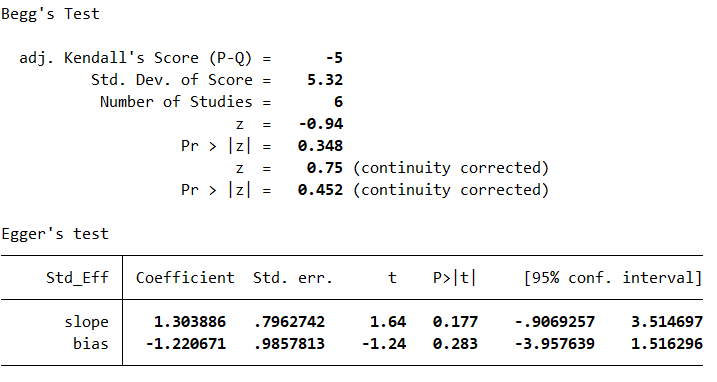
**

**Permanent vocal cord paralysis**

**
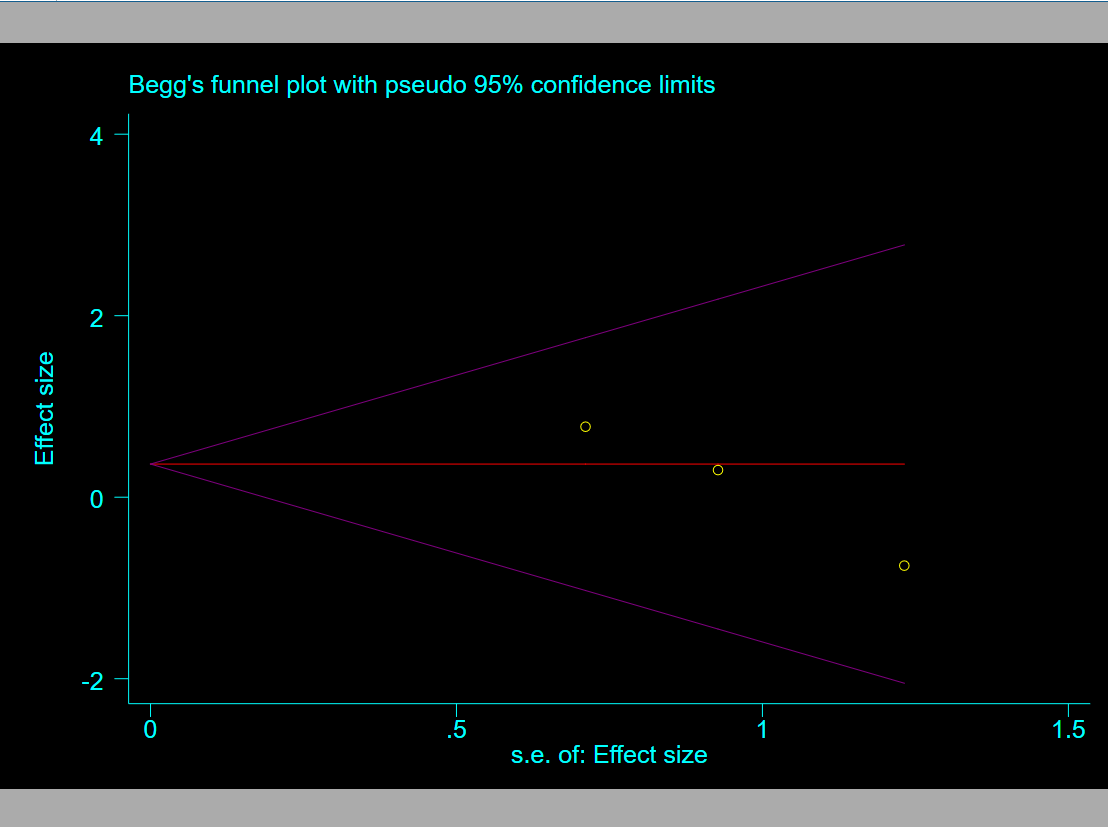

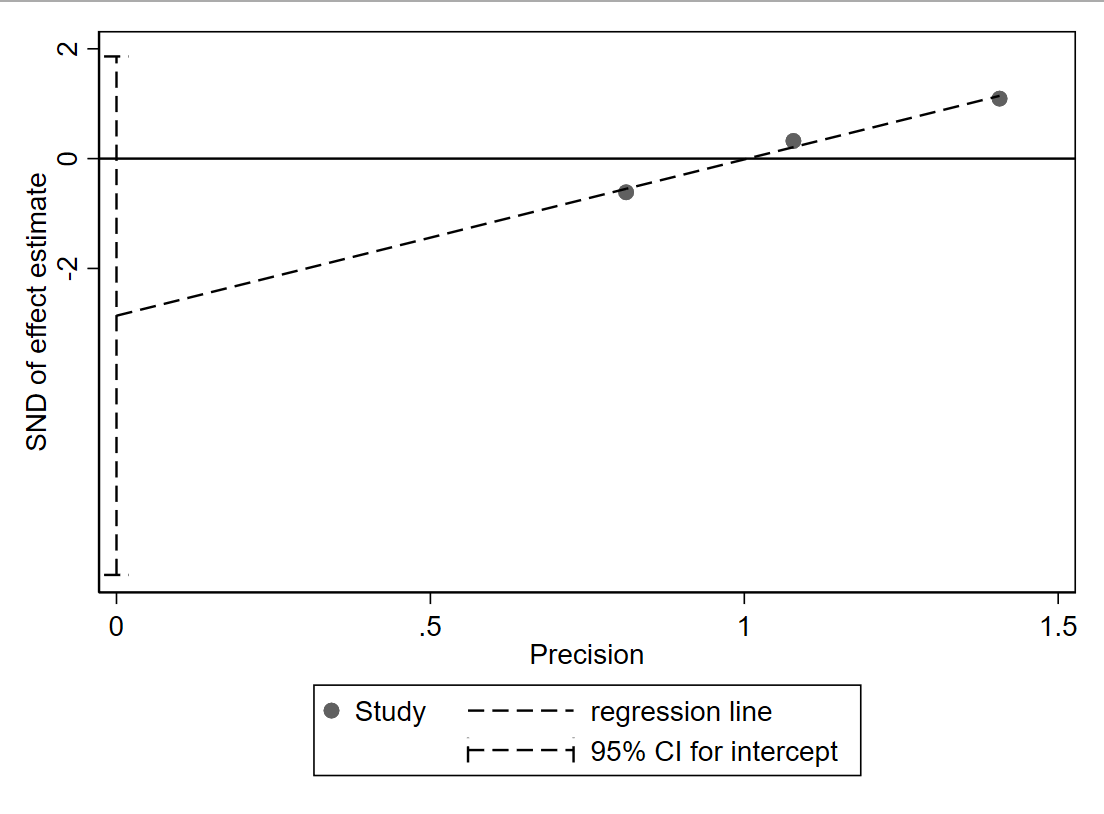

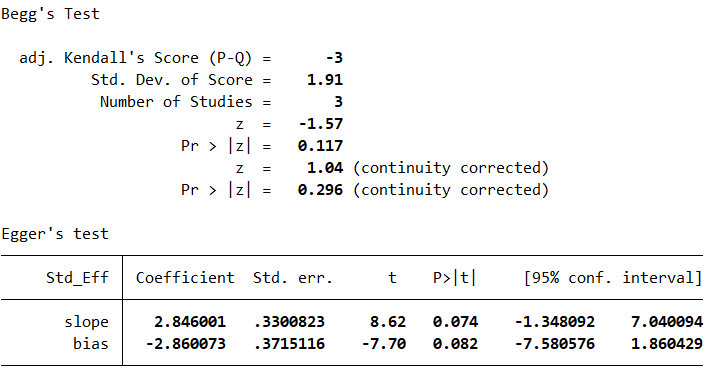
**

**Transient recurrent laryngeal nerve injury
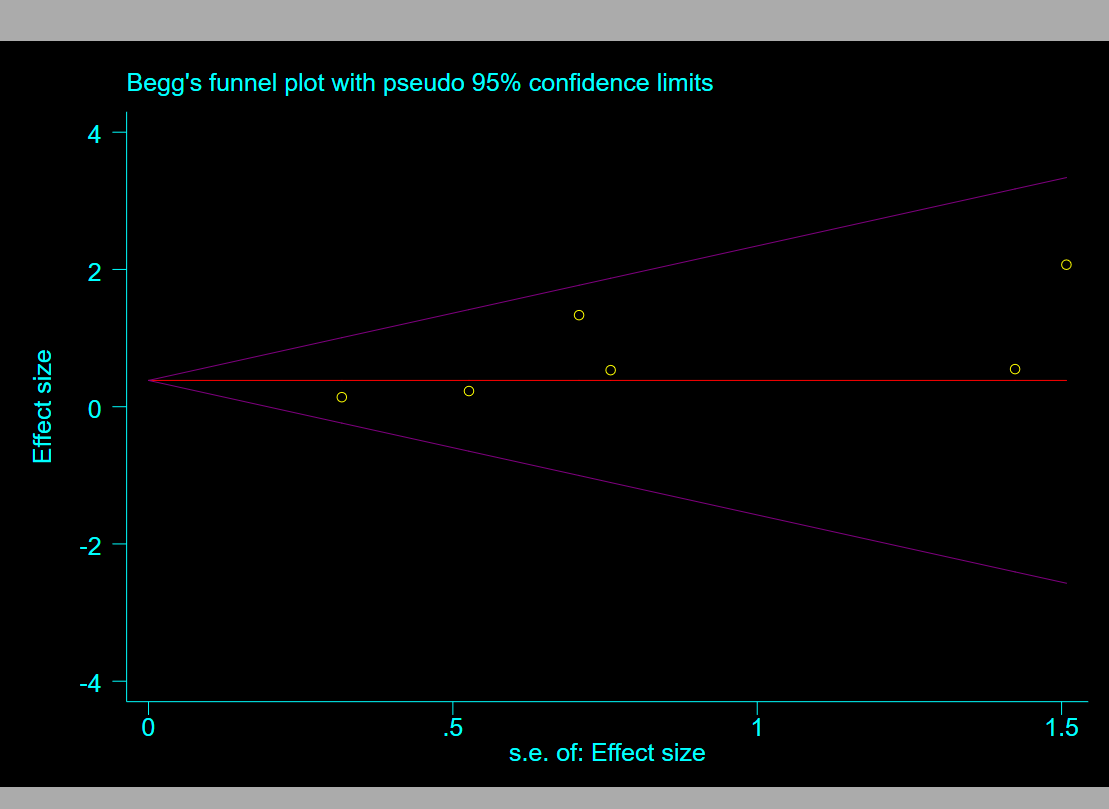

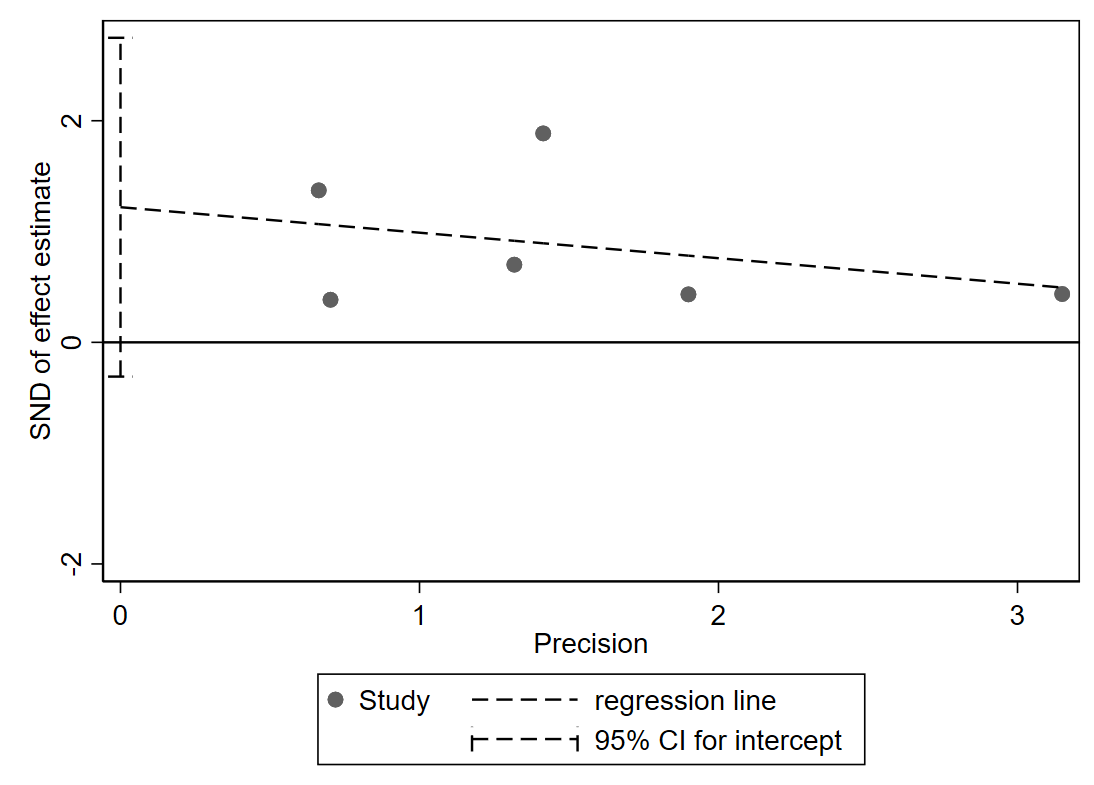
**

**
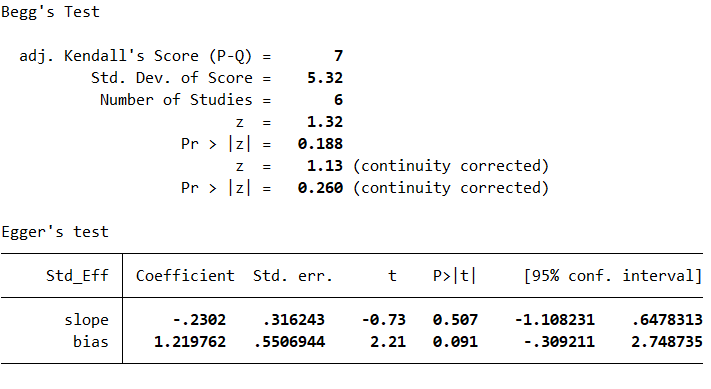
**

**Permanent recurrent laryngeal nerve injury**

**
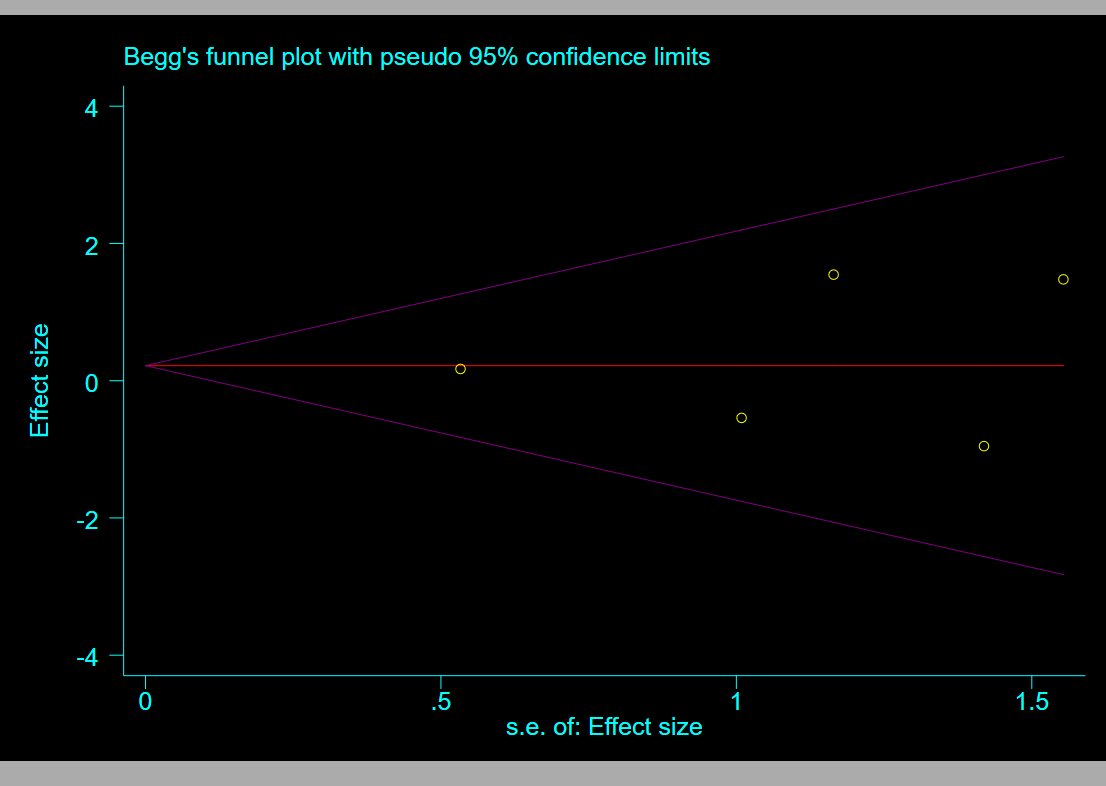

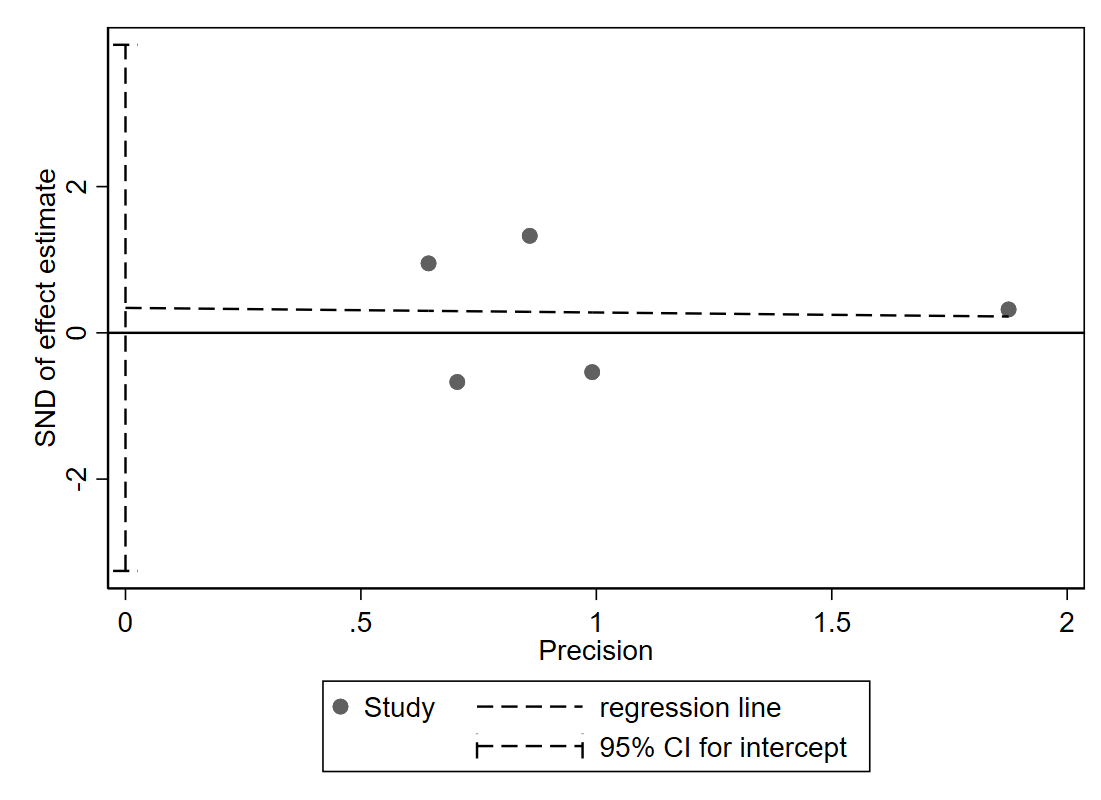

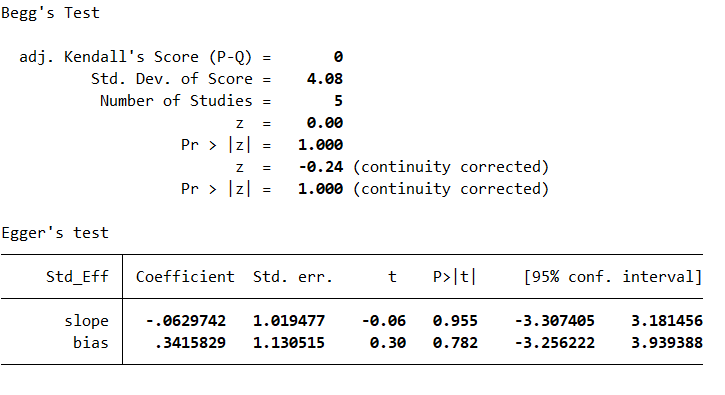
**

**Transient hypoparathyroidism**

**
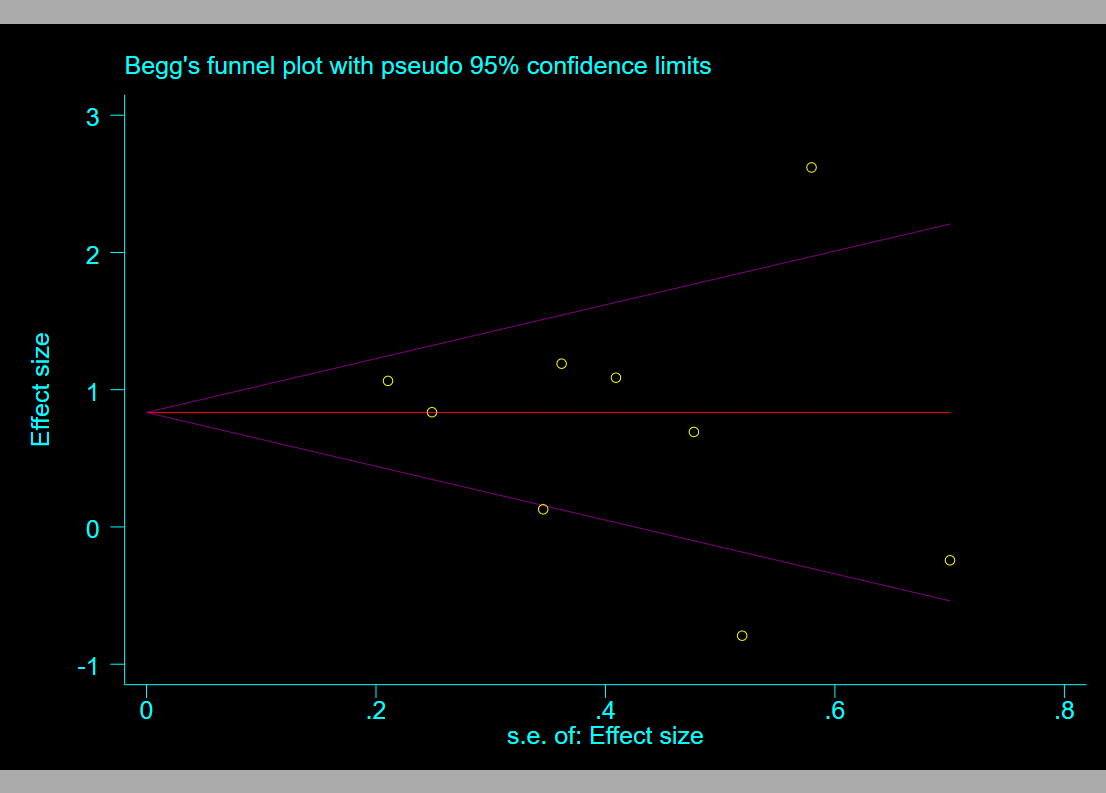

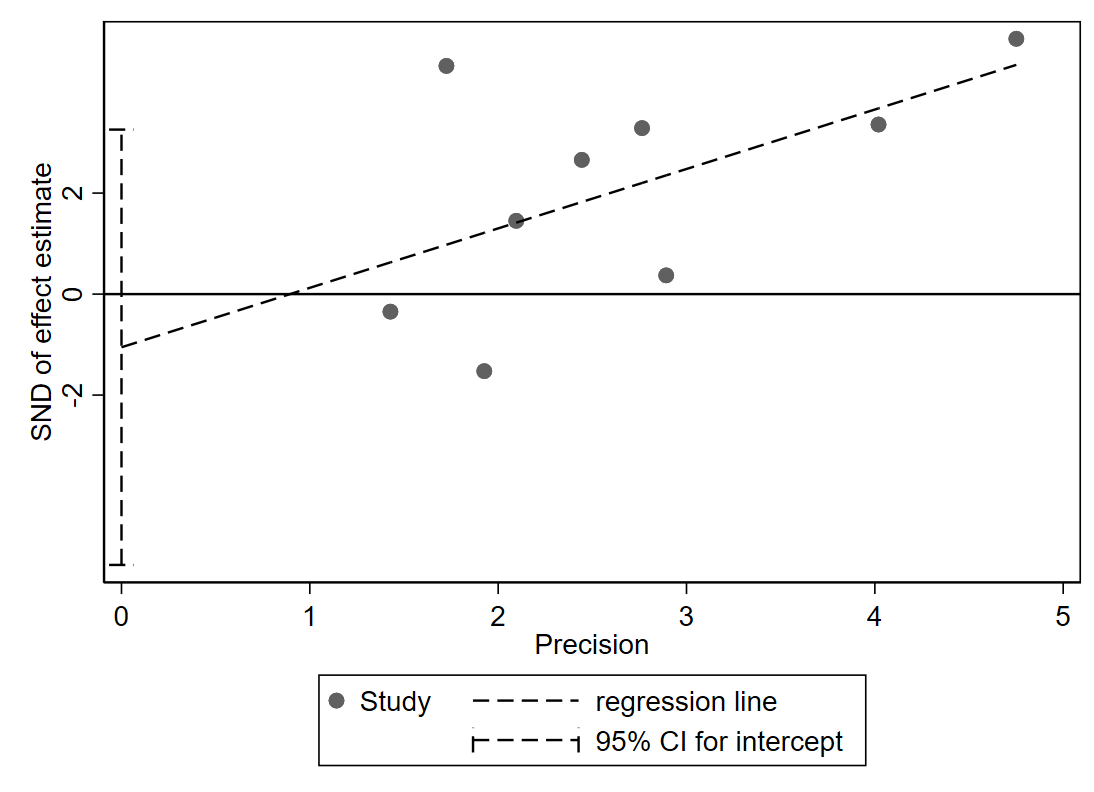

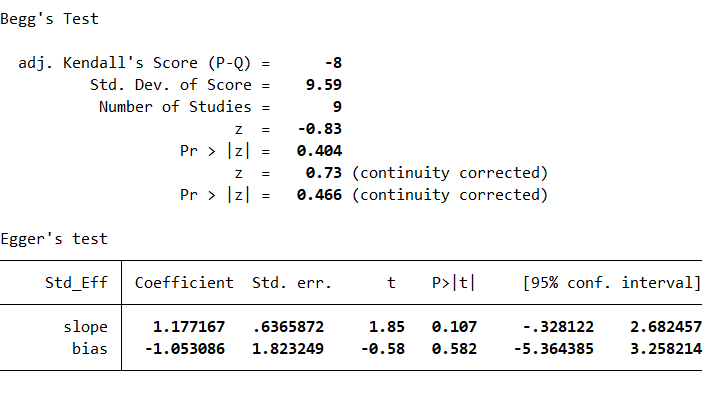
**

**Permanent hypoparathyroidism**

**
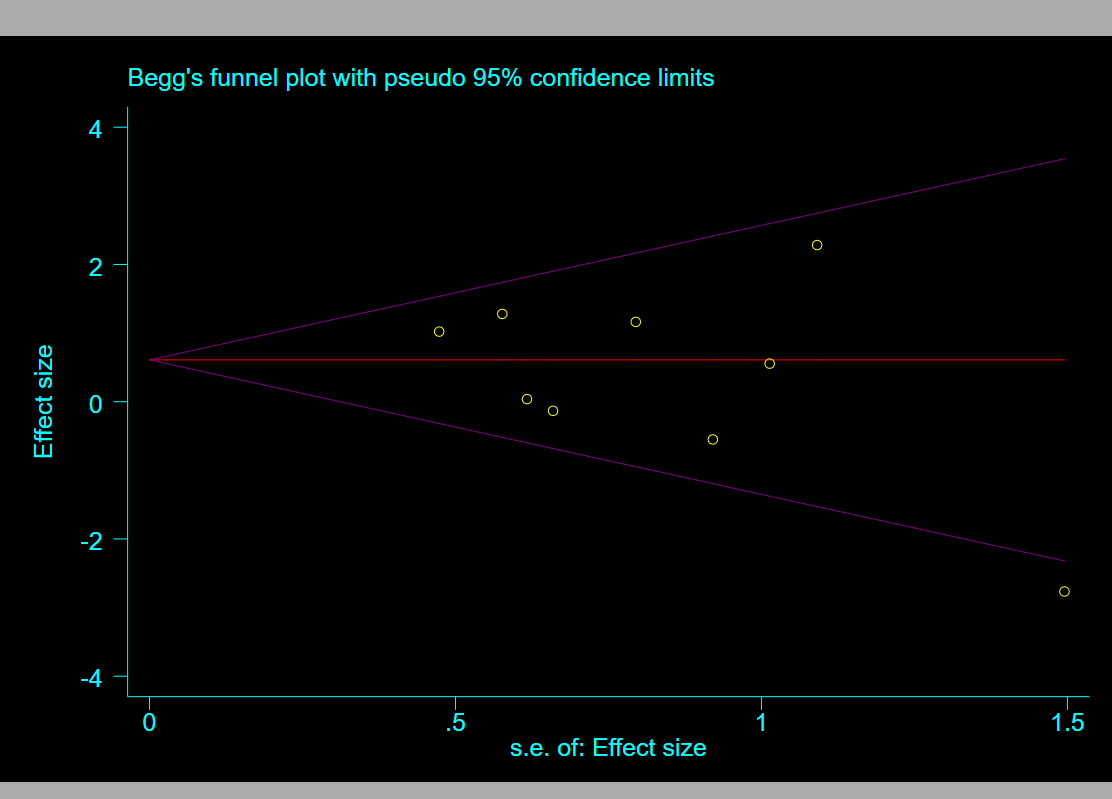

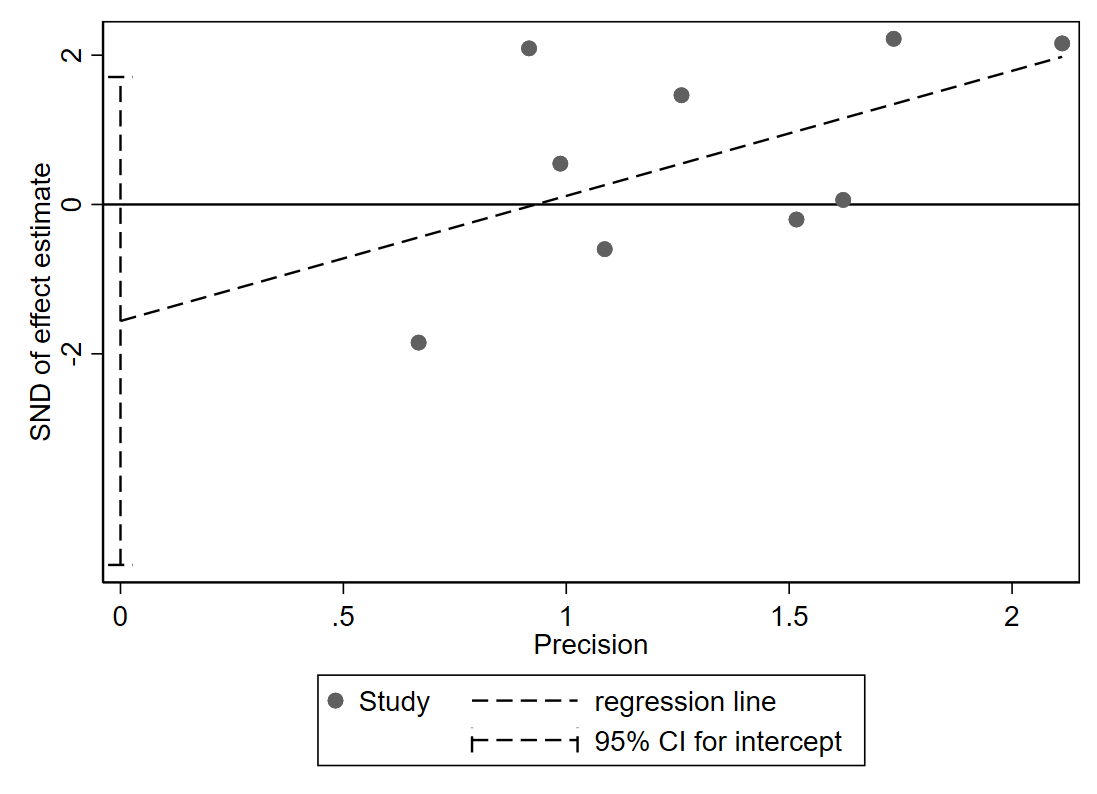

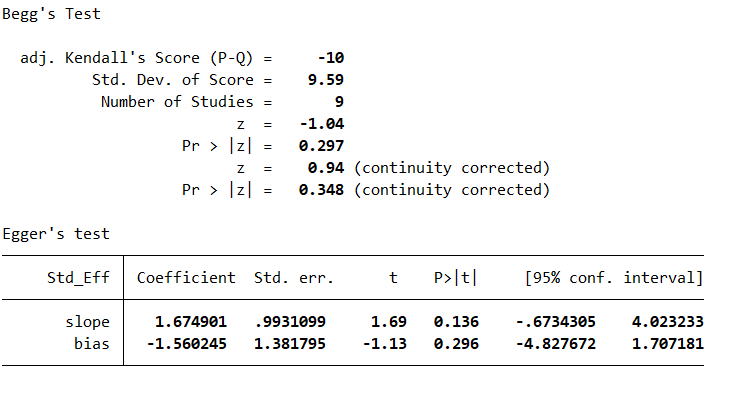
**
