# Supplementary material for: The effectiveness and safety of prophylactic central neck dissection in clinically node-negative papillary thyroid carcinoma patients: A meta-analysis
Source: Front Endocrinol (Lausanne). 2023 Jan 17;13:1094012. doi: 10.3389/fendo.2022.1094012 (PMC9886572; doi:10.3389/fendo.2022.1094012)
Supplement: Supplementary file 2 [file Table_1.docx]

**Supplementary Table 1.** The Newcastle-Ottawa Scale.

| Author | Country | Year | Selection | Comparability | Outcome | Score |
| --- | --- | --- | --- | --- | --- | --- |
| So YK | Korea | 2012 | **** | * | *** | 8 |
| Hyun SM | Korea | 2010 | **** | ** | *** | 9 |
| HartlDM | France | 2013 | **** | * | *** | 8 |
| Dobrinja C | Italy | 2017 | **** | * | *** | 8 |
| Jin SH | Germany | 2019 | **** | ** | *** | 9 |
| Moo TA | America | 2010 | **** | * | *** | 8 |
| Calò PG | Italy | 2016 | **** | * | *** | 8 |
| Yazıcı D | Germany | 2020 | **** | * | *** | 8 |
| Barcński M | Germany | 2013 | **** | * | *** | 8 |
| Conzo G | Italy | 2014 | **** | ** | *** | 9 |
| Korkmaz MH | Turkey | 2017 | **** | * | *** | 8 |
